# Dual-drug (Curcumin/Ciprofloxacin) loading and release from chitosan-based hydrogels embedded with magnetic Montmorillonite/Hyaluronic acid for enhancing wound healing

**DOI:** 10.1186/s13036-023-00385-1

**Published:** 2023-10-31

**Authors:** Zahra Sayyar, Gholam Reza Mahdavinia, Alireza Khataee

**Affiliations:** 1 Department of Chemical Engineering, University of Bonab, Bonab, 55513-95133 Iran; 2 Department of Chemistry, Faculty of Science, Polymer Research Laboratory, University of Maragheh, Maragheh, 55181-83111 Iran; 3 Department of Applied Chemistry, Faculty of Chemistry, Research Laboratory of Advanced Water and Wastewater Treatment Processes, University of Tabriz, Tabriz, 51666−16471, Iran; 4 Department of Environmental Engineering, Gebze Technical University, Gebze, 41400 Turkey

**Keywords:** Magnetic montmorillonite, Chitosan, Hyaluronic acid, Drug release, Wound healing

## Abstract

Montmorillonite (MMt) is extensively applied as an efficient drug-carrier in designing drug delivery systems (DDS) due to its high specific surface area to load drugs. Modification of MMt via iron (Fe) blending can thus be a desirable method to improve its biocompatibility. Herein, magnetic nano-carriers involving the magnetic MMt (mMMt) core surrounded by chitosan (Chito) as a biopolymer and hyaluronic acid (HA) were prepared. To coat the mMMt fabricated through the coprecipitation of the Fe^3+^/Fe^2+^ ions in the presence of MMt, the acquired mMMt as the core was then treated with the Chito/HA solution to induce the cross-linked Chito/HA as the shell (namely, the Chito/HA-mMMt). The transmission electron microscopy (TEM) results accordingly revealed the existence of the mMMt inside the Chito/HA solution. Curcumin (CUR) and ciprofloxacin (CIP) were further employed as two model drugs. The CUR and CIP release from the Chito/HA-mMMt subsequently occurred in a sustained manner and pH-dependently. Additionally, an upsurge in the CUR and CIP release by applying an external magnetic field was observed. Thus, the prepared Chito/HA-mMMt hydrogels promise an outstanding potential performance in terms of expanding novel pH-dependent DDS with a sustained release behavior. The scratch assay of the given hydrogels also confirms their applications for wound healing.

## Introduction

Hydrophilic polymers swell in aqueous solvents, and absorb and retain water without any destruction in their structure if there are chemical or physical bonds among their macromolecular chains. This type of polymer is typically identified as a hydrogel [1], with many functional groups in its structures, including three-dimensional (3D) hydrophilic polymers. The structure of the polymer network and some other parameters, such as pH, ionic strength, redox potential, chemical agents, temperature, light, ultrasound, as well as electric and magnetic fields, accordingly affect the amount of adsorbed water by this hydrogel [2, 3].

Hydrogels can be further utilized in diverse areas, such as wastewater treatment, drug delivery systems (DDS), tissue engineering, cosmetics, and agronomy. The hydrogel-based DDS has thus presented some advantages, viz., reduced side effects along with sustained and controlled drug release (DR), as compared with the formal ones. These targeted systems have attracted much attention among researchers because they help regulate DR, improve prescription dosage efficiency, minimize side effects of conventional methods, and even relieve chemotherapy-induced pain [4, 5]. Biopolymers also include three subsets of polysaccharides, polyesters, and proteins. In this line, chitosan, as a linear polysaccharide, is of utmost importance in biomedical and pharmaceutical delivery through polymerization with other polymers [6, 7] whose properties often vary depending on environmental stimuli, such as the pH variations [8]. Besides, the amino groups in the chitosan structure allow for more interactions with negatively charged molecules, and their protonation further leads to the chitosan dissolution in an acidic environment (pK_a_~6.5) [9, 10]. This feature in chitosan-based DDS seems to be effective in wound healing [11–13]. Moreover, the use of nano-carriers in such systems [14] together with wound [15] and burn dressings [16], and essential biomaterials in tissue engineering [17, 18] are other critical applications of chitosan, based on swelling capacity, oxygen permeability, blood coagulation, and antibacterial properties [19]. In order to physically modify and enhance some properties, a combination of chitosan with other materials, e.g., montmorillonite (MMt) and hyaluronic acid (HA) can be thus beneficial [20–22].

Over recent years, the mixture of two or more different polymers to produce hydrogels has been investigated because of their specific properties. Among the desired materials are the chitosan and hyaluronic acid blending with its unique features [23, 24]. As there is glycosaminoglycans in the cartilage structure which, chitosan has many structural similarities with glycosaminoglycans; therefore, chitosan can be exploited in cartilage tissue engineering [25, 26]. However, chitosan suffers from weak cell binding and growth capacity due to its hyper-hydrated structure, particularly in the hydrogel system [27, 28]. Moreover, there is HA in cartilage, which typically contains the repeating disaccharide units of N-acetylglucosamine and glucuronic acid (C_6_H_10_O_7_) [29]. As well, one of the main elements in the cartilage extracellular matrix is HA, characterized by a negative surface charge. It is thus predictable that HA plays a key role in repairing and building cartilage tissue because of the suitable interaction between HA and cartilage as well as the stem cells via the surface receptors [30].

As the main constituent of bentonite and hydrophilic mineral soil, MMt has been investigated for its structural domains and potentials, like swelling, cation exchange capacity, dispersibility, etc. to make it for use in medical applications with regard to its large specific surface area [31, 32]. However, MMt can be grafted into hydrogel to enhance its features in diverse areas, i.e., adsorption, sustained release, and reduced production costs [33–35]. Nowadays, some studies have established that chronic inflammation and bacterial infection are among the key reasons for postponed wound healing since they impede angiogenesis, the secretion of plasminogen activators, proteolysis enzymes, angiogenesis, and oxidative stress, as well as the accumulation of toxic materials [36, 37]. In this regard, ciprofloxacin (CIP) is one of the antibiotics that may be prescribed for killing both Gram-negative and -positive bacteria in wounds and accelerate wound healing [38]. Moreover, curcumin (CUR), an extracted natural polyphenol from turmeric, can act as an anti-inflammatory and antioxidant agent [39]. As evidenced in some studies, CUR is suitable for wound healing, endothelial dysfunction, and mucosal damage [40].

In this vein, Jafari et al. [41] demonstrated that the κ-carrageenan/chitosan/magnetic MMt (mMMt) hydrogels, including a high amount of mMMt, displayed the sunitinib maleate release of 64.0% and 8.6% at pH = 5.5 and 7.4, respectively. To the best of the authors’ knowledge, synthesizing Chito/HA hydrogel with mMMt to load antibiotics, such as CIP and CUR, has not yet been fulfilled.

Using an external magnetic field, a drug can be accordingly directed to the targeted tissues in a magnetic DDS, which leads to highly efficient drug release [4].

In this study, a Chito/HA-based hydrogel was synthesized using a conventional technique to enhance release performance. Moreover, acetic acid was recruited to act as a cross-linking agent. Upon cross-linking, free carboxyl (–CO_2_H) groups were accessible, which could be deprotonated in the neutral and alkaline media, thereby creating partially negatively charged particles [42]. Furthermore, it was expected that the mMMt grafting in these hydrogels would prompt their properties. Nanocomposite hydrogels (namely, Chito/HA-mMMt) were further synthesized via the freeze-thawing technique. These hydrogels, as the pH-responsive ones, could rapidly reply to the external changes in pH and release drugs at the desired spots. Therefore, loading drugs on chitosan would increase using MMt as a nano-clay. Dual-drugs, i.e. CIP/CUR, as two drug models, were then loaded on the Chito/HA-mMMt hydrogels (viz., drug-loaded Chito/HA-mMMt) to examine their release, wound healing efficiency, cytotoxicity assay, and antibacterial activities. The present study accordingly concentrated on drug release in two media, i.e., the simulated acidic pH = 5.5 and 7.4. Moreover, the release mechanism was investigated using the Higuchi and Korsmeyer-Peppas models.

## Materials and methods

### Materials

Ciprofloxacin hydrochloride (Sigma-Aldrich Chemical Co., USA) and Curcumin (97%, Merck Co., Darmstadt, Germany) were applied as two drug models to load in the hydrogels. Natural sodium montmorillonite was also purchased from Southern Clay Products, USA. Dimethyl sulfoxide (DMSO), Chitosan (M_w_= 50 ± 10 kDa, and 85% of acetylation), and agar were further obtained from Sigma-Aldrich Chemicals Co., USA. As well, acetic acid (CH_3_COOH) and citric acid (CA, HOC(CO_2_H) (CH_2_CO_2_H)_2_), were obtained from Merck Co. (Darmstadt, Germany). Hyaluronic acid sodium was also purchased from Shangdong Freda Biochemical Industry Co., (China, HA-EP2000).

Moreover, Dulbecco’s modified eagle medium (DMEM) and fetal bovine serum (FBS) were acquired from DENAzist Asia Co., Iran. Furthermore, normal human fibroblastic cells (L929) were purchased from Pasteur Institute of Iran. To evaluate the antibacterial activities, both Gram-positive *Staphylococcus aureus* (*S. aureus*) and Gram-negative *Escherichia coli* (*E. coli*) bacteria (from NIGEB Bacterial Bank, Iran) were applied. During all processes, deionized (DI) water was only used. The rest of the chemical substances utilized for this purpose were in analytical grades and without extra purification.

### Magnetic-montmorillonite (mMMt) preparation

The mMMt nanoparticles (NPs) were created using an improved coprecipitation technique. To synthesize such NPs, it was thus necessary to recruit a stoichiometric ratio of 1:2 of Fe (III) sulfate: Fe (III) chloride (Fe_2_SO_4_:FeCl_3_), which was dissolved under nitrogen (N_2_) atmosphere to prevent oxidation, while being stirred at 70 °C in DI water. Then, 1 g MMt was poured into 100 mL of DI water, and dispersed with ultrasonic for 20 min at the frequency of 50 kHz (Bandelin SONOPULS HD 2200) to combine it with the aforementioned solution. Chemical precipitation was also gained by adding the ammonium hydroxide (NH_4_OH) solution (25%) drop by drop under vigorous stirring to achieve pH = 10. Following this reaction, the dark orange color of the solution directly turned black, and stirring continued for 1 h. Eventually, the Fe_3_O_4_/MMt NPs were created. The black magnetic precipitates accordingly needed to be washed numerous times with fresh distilled water to obtain a solution with pH = 7. For this purpose, an external magnetic field was used to accumulate the sediments at the bottom of the container.

### Preparation of chitosan/hyaluronic acid-mMMt nanocomposite hydrogels (Chito/HA-mMMt)

Chitosan solution was provided by dissolving 1 g of chitosan powder in 50 mL acetic acid solution (1 wt%) by a stirrer at 70 °C. The transparency of the solution meant the completion of the chitosan dissolution process. Through the coating of chitosan onto the mMMt NPs the mMMt amount was optimized to reach the highest efficiency. In another beaker, different amounts of dried mMMt-NPs (namely, 0.16 and 0.32 g) were added to 50 mL of DI water, and then sonication at 50 kHz (Bandelin SONOPULS HD 2200, set to 40% of total 100 watts) was utilized to disperse mMMt for 10 min. After that, 0.1 g of HA was poured into this aqueous solution, and then placed on the stirrer at 40 ºC for about 1 h to obtain a uniform solution. To prepare the mMMt-hyaluronic acid/chitosan nanocomposite hydrogels (Chito/HA-mMMt1 and Chito/HA-mMMt2 hydrogels), the chitosan solution was added into the mMMt one at 40 ºC, and then stirred for 1 h. In the next step, 1 g of citric acid was dissolved in 10 mL of distilled water and subsequently added to the solution and stirred at 70 ºC for 4 h to ensure adequate interactions between positively charged chitosan molecules and the negatively charged mMMt ones, fastened at the external surface of Fe_3_O_4_. It took more than 4 h to complete the cross-linking operation. Lastly, the desired hydrogel was synthesized using the freeze-thawing method three times. In the course of this technique, the hydrogels were frozen at -18 ºC and kept at this temperature for 12 h. Then, the frozen hydrogels were placed at 25 ºC to thaw for 5 h. In order to achieve a highly porous hydrogel, they were further lyophilized using a freeze-dryer (Alfa 2-4LDplus, Christ Co., Germany).

### Drug loading/release

The nanocomposite hydrogels to load drugs were prepared using the technique described above. The difference was that for loading CIP and CUR into the Chito/HA-mMMt, 5 mL of the CIP solution and 5 mL of CUR (0.5 mg/mL) were poured into a dispersion of the mMMt/HA solution, then other steps were continued as mentioned earlier.

The ultraviolet-visible (UV-Vis) spectrophotometry at λ_max_ = 275 and 425 nm, and the calibration curve were then applied to measure the amount of CIP and CUR in the media, respectively. To examine the drug release, drug-loaded samples were placed in two media, i.e., neutral (pH = 7.4) and acidic (pH = 5.5), separately. As well, 0.05 g of hydrogels containing dual-drugs was immersed into 50 mL of these media. A rotary shaker (90 rpm) was further recruited to investigate the release at 25 ºC. To measure the percentage of the released CIP and CUR, at definite time intervals, 2 mL of buffer was also selected, and 2 mL of the new solution was returned in order to maintain a constant volume of the media. Equation 1 was further employed to measure the drug release percentage (DR%). All tests were accordingly performed twice, and their average was presented.

1 $$DR\left(\%\right)=\frac{\left(VC_n{\displaystyle+\sum_{i=1}^{i=n-1}}V_iC_i\right)}{M_0}\times100$$

whereinwhere, *V*, *V*_*i*_, *M*_*0*_, *C*_*n*_, and *C*_*n−1*_ refer to are the total volume of the release media, volume at each time interval, the amount of drug loaded in the hydrogels, and the concentrations in the releasing media after the *n* and *n-1* withdrawing steps, respectively.

### Cell viability assay

The L929 cells, as the normal human fibroblastic cells, were chosen here to examine the cytotoxicity effect of mMMt. They were thus grown in the DMEM (10% FBS and 1% Penicillin–Streptomycin, 100 U/ml) at the standard condition of culture. The cells were further seeded in a 96-well plate and incubated at 37 ºC for 24 h. After the incubator time, the cells were treated with pure CIP, pure CUR, and dual-drug (CIP/CUR)-loaded Chito/HA-mMMt, separately. Subsequently, 200 µL of the 3-(4,5-dimethylthiazol-2-yl)-2,5-diphenyl tetrazolium bromide (MTT, blue-violet crystals) solution (5 mg/mL) was added into the wells and then incubated at 37 ºC for 4 h. Finally, 200 µL of DMSO was poured into the wells to disband the formazan crystals shaped by the living cells. A microplate reader (Elx808, BioTek, USA) was further applied to estimate the absorbance at 570 nm. All tests were done twice times to minimize the errors. The ratio of the treated cells to the untreated controls accordingly displayed the cell viability percentage (Eq. 2).

2 $$Cell\;viability\;(\%)=\frac{Absorbance\;of\;treated\;cells\;at\;570\;nm}{Absorbance\;of\;untreated\;cells\;at\;570\;nm}\times100$$

### Antibacterial test

To examine the antibacterial activities of the samples, both bacteria, viz., *S. aureus and E. coli*, were applied through the hole diffusion technique. In this regard, the nutrient agar culture media were treated by 0.5 McFarland turbidity standards of bacterial activities (1.5 × 10^8^ colonies) at the plates (10 cm in diameter). Afterward, a disc of the drug and 0.1 g of each hydrogel were placed in the hole on the plate. The organized plates were also incubated at 37 ºC for 24 h. Finally, the antibacterial activities of the samples were depicted by generating a clear zone area around the hole.

### Swelling measurement

One of the unique properties of hydrogels is the swelling factor (SF), which means the amount of water they can adsorb. To measure the SF, 0.1 g of dried hydrogel was dipped into water and the buffer solutions with pH = 5.5 and 7.4, and got wet for 24 h. The extra surface water was also removed by tissue paper. The swelled hydrogels were then weighted, and the SF was calculated using Eq. 3:

3 $$SF\;\left(g/g\right)=\frac{W_s-W_d}{W_d}$$

wherein *W*_*s*_ and *W*_*d*_ represent the weights of the swelled and dry samples, respectively. To achieve more precise results, all tests were performed three times, and their average was reported.

### Wound healing

A scratch wound analysis was applied to display the diffusion and migration abilities of the Swiss 3T3 fibroblasts, which could estimate the growth of the cell population on the surfaces. The 24-well tissue culture plates, including coverslips pre-coated with collagen type I (40 mg/ml), were accordingly employed to seed the cells for 3 h at the temperature of 37 ºC, and 3 × 10^5^ cells per ml. The cell monolayers were further cultured in the medium containing 10% FBS. After that, a sterile plastic micropipette tip was utilized to create a linear gap between the cells in the monolayer. Moreover, phosphate buffer saline (PBS) was applied to wash the coverslips and remove any cellular debris. The Chito/HA-mMMt2 (10 mg/ml) was consequently added to this culture, and incubated for 12 h at the temperature of 37 ºC in the presence of carbon dioxide (CO_2_). Notably, the experiments were repeated twice.

### Physicochemical characterization

The morphology of the hydrogels was characterized by the transmission electron microscopy (TEM) and the field emission scanning electron microscopy (FE-SEM), using the Philips EM 208 S operating at 100 kV and a Tescan MIRA3 microscope (the Czech Republic), respectively. The energy-dispersive X-ray (EDX) spectroscopy of the samples was also performed with the Zeiss Sigma 300. As well, the X-ray diffraction (XRD) was applied to study the construction of nanocomposites by the Tongda-TD-3700, China, using the Copper K-alpha (CU-Kα) radiation (λ = 0.15418 nm) operating at 30 kV and 20 mA from 10 to 80 degrees. In order to detect the behavior of the hydrogels against heat, the thermo-gravimetric analysis (TGA, STA6000, Perkin-Elmer, USA) was done. Moreover, the Fourier transform infrared spectrometry (FT-IR) spectra were obtained using the Tensor 27, Bruker, Germany, and the KBr pellet method in the range of 400–4000 cm^−1^ was practiced to identify the structure, composition, and the structural changes of different hydrogels. To measure the zeta potential values, the dynamic light scattering (DLS) analyzer (SZ-100z, Horiba Jobin Jyovin Co., Japan) was recruited. The magnetic behaviors of the hydrogels were accordingly studied by a vibrating sample magnetometer (VSM, Model 7400, Lakeshare Company, USA) with an applied field range of -9000 to 9000 O_e_ at 298 K. Besides, a freeze-dryer (Alfa 2-4LDplus, Christ Co., Germany) was used to dry and pulverize the samples. The adsorption was further recorded using the UV-Vis spectrophotometer (viz., double-beam Shimadzu UV-1800, Japan).

### Statistical analysis

Statistical analysis of the obtained results was carried out using SPSS software version 16.0. Data were shown as mean ± standard deviation (SD) at a significance level of ρ<0.05.

## Results and discussion

### Synthesis and characterization of hydrogels

**Fig. 1:**
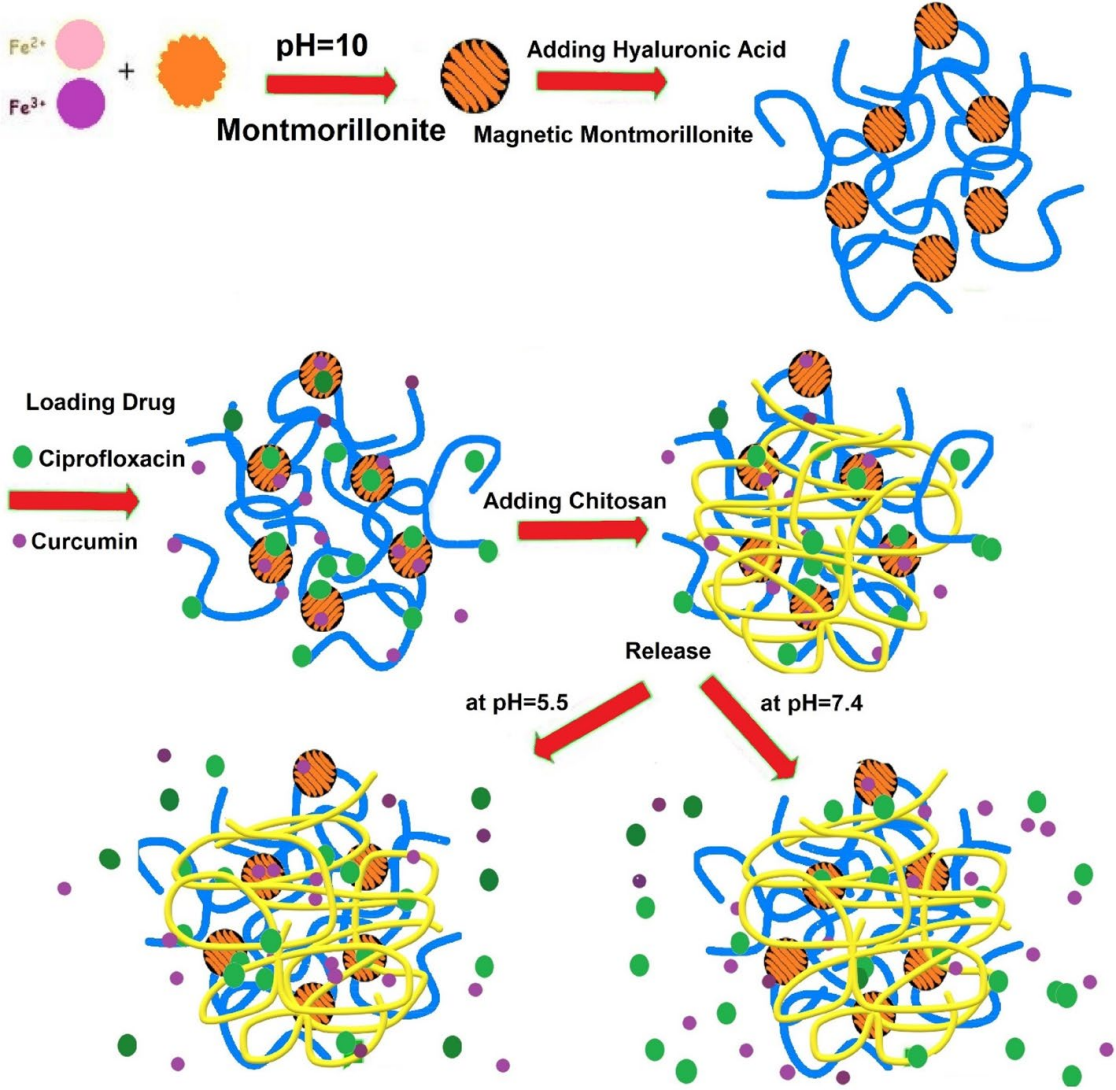
A simple scheme giving the process to synthesize Chito/HA-mMMt nanocomposite hydrogels

To construct the chitosan-based magnetic hydrogel nanocomposites, magnetic montmorillonite nanoparticles were first synthesized through in-situ co-precipitation or the Fe^2+^/Fe^3+^ ions in the presence of the mMMt nano-clay. The adsorption of the Fe ions on the anionic centers of MMt and the intercalation between the MMt layers accordingly resulted in the formation of the magnetic NPs not only on the clay surface but also between the MMt layers. To reach a cross-linked magnetic chitosan-based hydrogel nanocomposite, the purified mMMt was then transferred into the HA solution. Furthermore, citric acid (CA) as a cross-linker was added to chitosan, forming the cross-link points through the ester or amino functional groups. During the cross-linking reaction, the mMMt NPs and HA were in the CA-chitosan hydrogel nanocomposites. A simple scheme, including the synthesis of the magnetic Chito/HA-mMMt nanocomposites hydrogels, is illustrated in Fig. 1.

#### FT-IR study

**Fig. 2:**
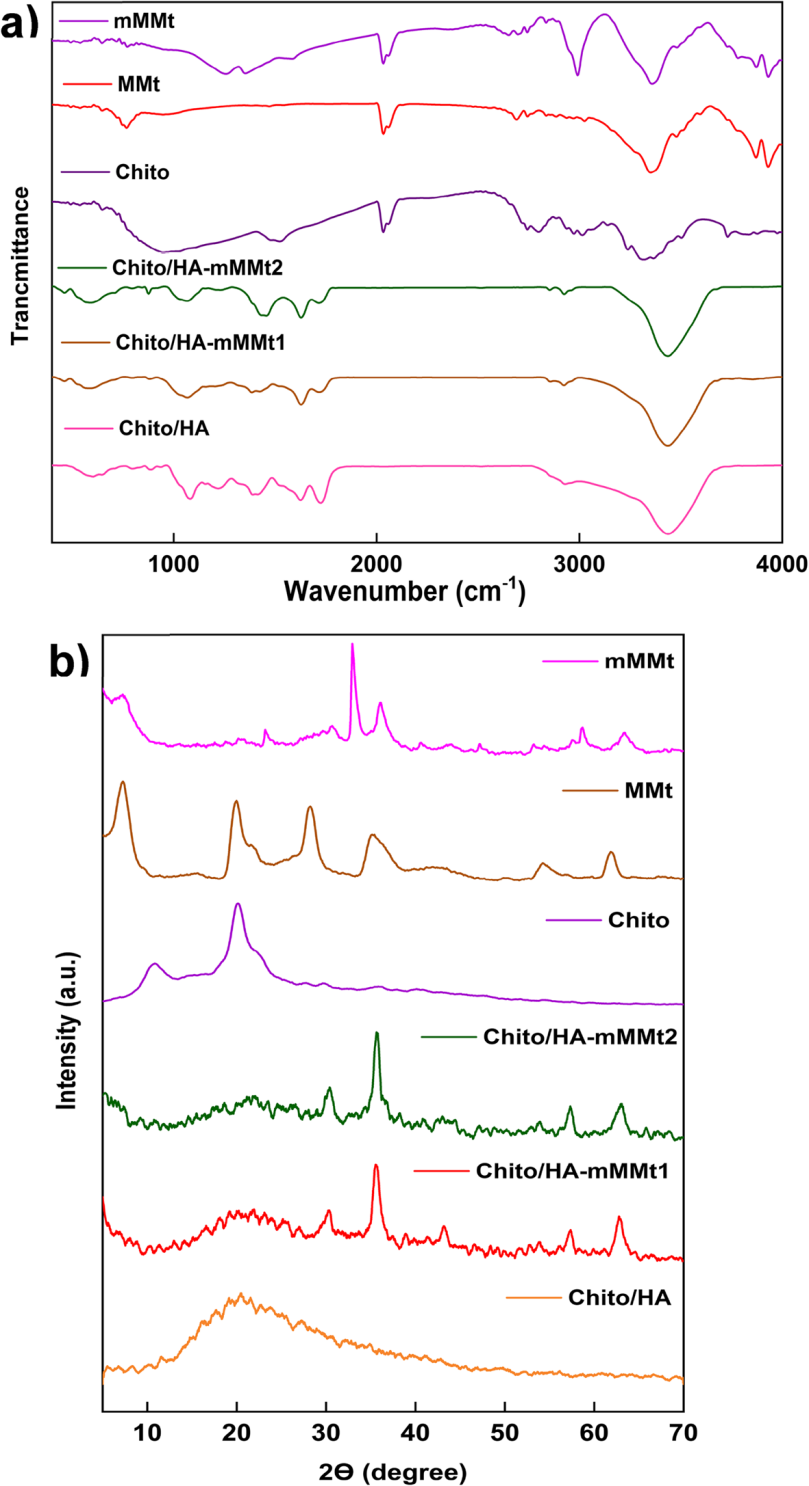
**a** FTIR spectra and** b **XRD patterns of raw materials and synthesized hydrogels

The possibility of cross-linking through the ester formation, originating from the reaction between the hydroxyl (–OH) groups on chitosan and the –CO_2_H groups of CA, was examined by the FT-IR, whose results are displayed in Fig. 2a. In the FT-IR spectrum of MMt, the broad peak at 3626 cm^−1^ was related to the stretching vibration of the –OH groups on the MMt clay, accompanied by the stretching vibration of the Al-O and the functional group of MMt. The stretching vibration modes of the Si-O-Al and Si-O-Si groups also had distinguishing peaks at around 794 and 1042 cm^−1^, respectively. The peaks appearing at 522 and 460 cm^−1^ were further associated with the bending vibration modes of the Si-O-Al and Si-O-Si groups, respectively [43]. In the chitosan spectrum, an overlapped and broad peak at 3443 cm^−1^ owing to the N-H and O-H groups was further observed. The appearance of the characteristic bands at 1697 and 1599 cm^−1^ in the chitosan spectrum could be thus assigned to the amide I and II stretches. As well, CA exhibited the IR peaks at 3370 and 3030 cm^−1^, attributing to the stretching vibration of the O-H and C-H bonds, respectively [44]. The symmetric and asymmetric stretches of the –CO_2_H groups on CA were also evident from the peaks in the FT-IR spectrum, appearing at 1398 and 1568 cm^−1^, respectively. Considering the FT-IR spectrum of the mMMt, the characteristic peaks of MMt at 1636 cm^−1^ (to 1328 cm^−1^) and 1042 cm^−1^ (to 1034 cm^−1^) were relatively shifted to the lower frequency, possibly due to the interaction between MMt and the immobilized Fe_3_O_4_-NPs [45]. The Fe_3_O_4_ IR peaks at around 400–600 cm^−1^ also overlapped with the MMt ones, so they were not detectable. The FT-IR spectra of hydrogel further showed the characteristic peaks of chitosan, Hyaluronic acid, mMMt, and citric acid. The intensity of all peaks for Chito-HA was thus higher than that of the Chito/HA-mMMt1 and Chito/HA-mMMt2. In the Chito/HA-mMMt2 sample, the peak at 1750 with low intensity also appeared, mostly assigned to the –CO_2_H groups of CA. Any peak related to the ester or amide formation due to the reaction between chitosan and CA was not visible. The overlapping of the IR peaks of the ester-amide functional groups with those of other ingredients might be thus responsible for the non-appearing new peaks.

#### XRD study

Figure 2b shows the XRD pattern of the MMt, mMMt, Chito/HA, Chito/HA-mMMt1, and Chito/HA-mMMt2. The XRD pattern of the MMt clearly revealed its distinctive peak around 2θ = 7.5, 20, and 28.1 degrees. Upon the immobilization of the Fe_3_O_4_-NPs on the MMt plates to reach mMMt, the characteristic MMt peak disappeared, possibly associated with the exfoliation of the MMt plates. This might be further attributed to the cation exchangeability of MMt in which the Fe ions could be intercalated between the MMt layers, and subsequently exfoliate by making the Fe_3_O_4_-NPs on the MMt layers. The creation of the Fe_3_O_4_-NPs in the presence of MMt was further confirmed by the diffraction peaks of (220), (311), (400), (422), (511), and (440) at 2θ = 30.0, 35.5, 43.2, 53.5, 57.0, and 62.6º, respectively. The XRD pattern of the neat chitosan powder also displayed two distinctive peaks at 2θ = 10.5 and 20.1º, which were related to the semi-crystalline structure of chitosan and hydrate, and the increasing hydrogen bonding and flexibility of the chitosan chain, respectively [41].

The crystallinity was the outcome of the presence of intermolecular hydrogen bonding in chitosan. These peaks demonstrated a regular structure formed as a result of hydrogen bonds between the amine and –OH groups, which were responsible for preventing chain movement. After the cross-linking of the Chito/HA-mMMt mixture with CA, the intensity of the chitosan peaks diminished, indicating the presence of amorphous chitosan in the composition of the nano-carriers. In the nano-carrier patterns, the presence of the Fe_3_O_4_-NPs was further established by the characteristic peaks at 2θ = 35.7 and 63º. Considering the high content of the mMMt, the intensity of the Fe_3_O_4_ peaks in sample 2 was sharper as compared with sample 1.

#### Morphology studies

**Fig. 3:**
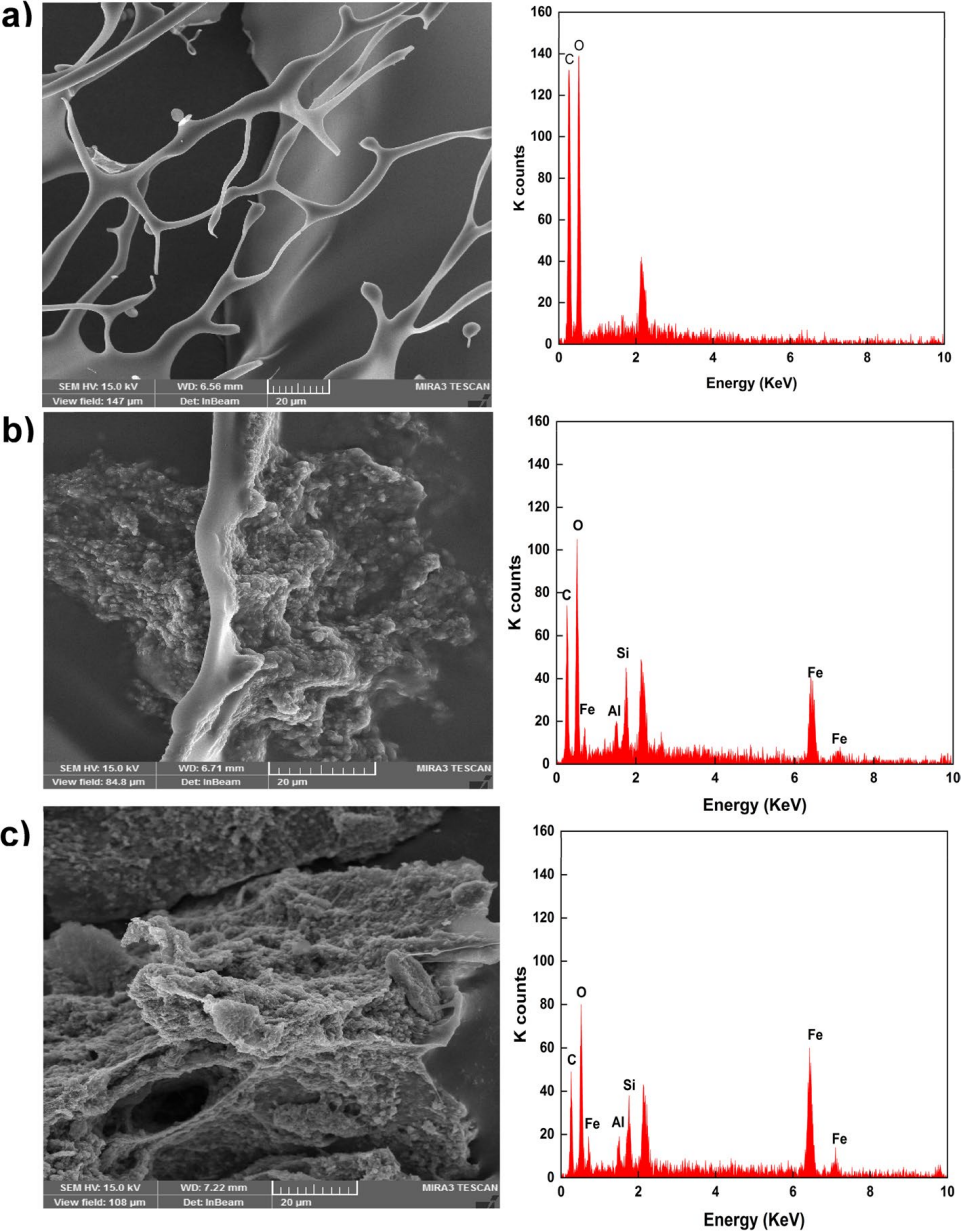
SEM and EDX image of **a** Chito/HA, **b** Chito/HA-MMt1, and **c** Chito/HA-MMt2

The SEM analysis was done to evaluate the influence of the mMMt on the surface morphology of the magnetic carriers and the hydrogel structure. Figure 3 shows the morphology of the hydrogels using SEM. It became apparent that all hydrogels have a porous structure.

Compared with the Chito/HA-mMMt hydrogels, the structure of the Chito/HA-mMMt1were more porous, and the size of the pores increasedincreases with the growth in the mMMt content. Moreover, these synthesized hydrogels had excellent interactions between chitosan, HA molecular chains, and the mMMt NPs.

The SEM image of the Chito/HA-mMMt also showed a rough surface with spherical particles. The corresponding formed particles could be accordingly ascribed to the magnetic NPs. According to this image, the size of the NPs was small. When the mMMt was introduced into the Chito/HA hydrogels to produce magnetic nano-carriers, a rough surface with spherical particles appeared with a larger size. The results might be devoted to the covering effect of the cross-linked Chito/HA on the mMMt, leading to a rise in the NPs size. Accordingly, the spherical NPs on the Chito/HA-mMMt2 were much more compared with the Chito/HA-mMMt1. The mMMt content utilized to prepare the Chito/HA-mMMt2 was also higher than that of the Chito/HA-mMMt1, and subsequently, the chitosan ratio dwindled. In other words, the low content of the mMMt in the Chito/HA/mMMt1 was embedded in the cross-linked chitosan carrier, and the spherical particles tended to drop.

The elemental analysis of the samples was further investigated by the EDX spectroscopy. In this line, the EDX of the mMMt displayed the Si, Al, and Ca elements, which were in agreement with previous research. In addition to the main MMt elements, the appeared Fe peak indicated the presence of the magnetic Fe_3_O_4_-NPs. In the EDX of the magnetic chitosan-based nano-carriers, the C and N peaks along with the mMMt elements, also appeared because of chitosan and HA ingredients.

#### TEM study

**Fig. 4:**
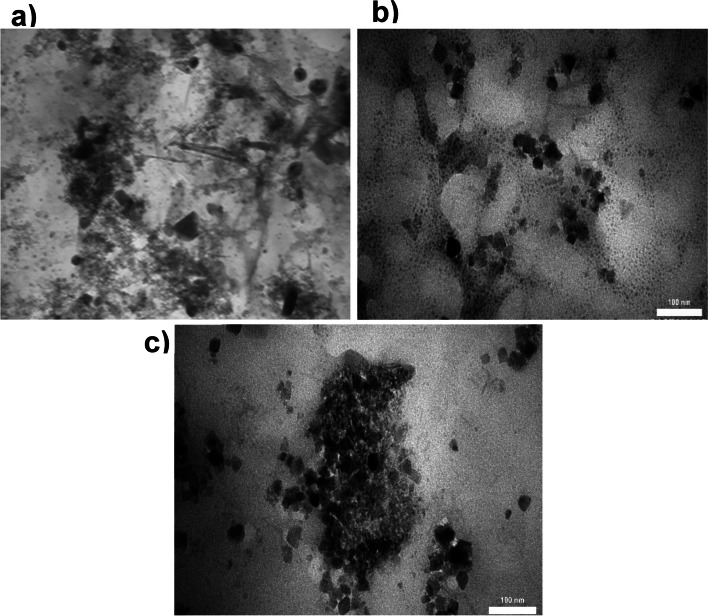
TEM image of **a** mMMt, **b** Chito/HA-MMt1, and **c** Chito/HA-MMt2

Figure 4 displays the TEM images of the mMMt, Chito/HA-mMMt1, and Chito/HA-mMMt2. According to the mMMt image, the MMt clay existed in the exfoliated platelets or intercalated tactoids. The formed Fe_3_O_4_-NPs could be further seen on the surface or among the MMt plates. By adding the mMMt to the chitosan/HA solution to generate the magnetic carriers, the mMMt-NPs were dispersed inside the chitosan/HA, resulting in the covering of the mMMt NPs by the chitosan ingredient. Unlike the mMMt, the MMt plates did not clearly appear that could be related to the interweaving of the chitosan chains into the MMt layers to produce the exfoliated MMt clay [46].

#### VSM study

**Fig. 5:**
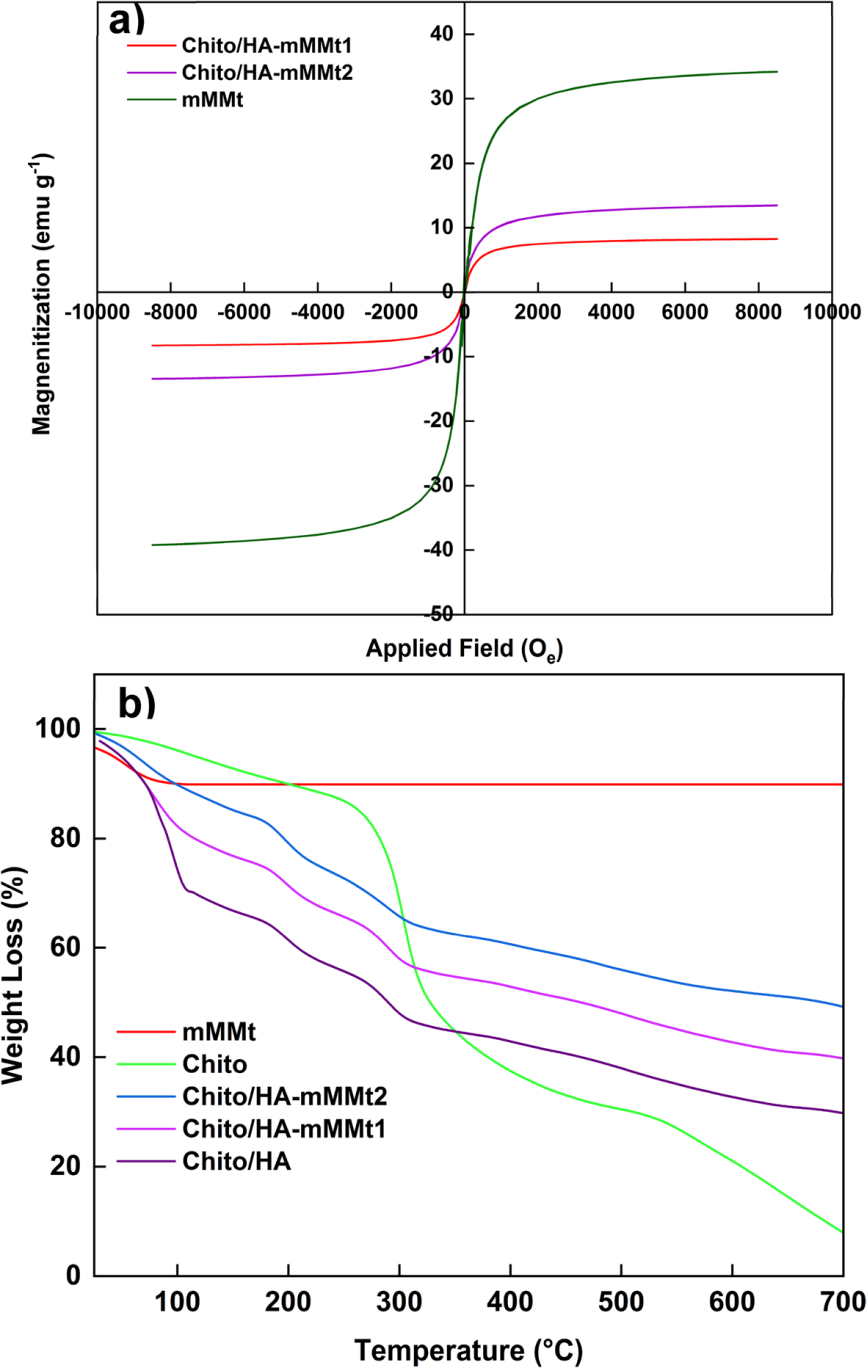
**a** Hysteresis loops and **b** TGA thermograms of raw materials and hydrogels

The magnetization behavior of the samples was investigated using the VSM technique at an applied field of ± 10 kOe at 298 K to compare the saturation magnetization variation (SMV) by different contents of the mMMt. The SMV of the mMMt, Chito/HA-mMMt2, and Chito/HA-mMMt1 were accordingly about 34.2, 13.4, and 8.2 emu/g, respectively (Fig. 5a). The magnetization curves of the mMMt, Chito/HA-mMMt1 and Chito/HA-mMMt2 also showed their superparamagnetic property with no remanence or coercivity [47]. Compared with the mMMt, the SMVs of the magnetic samples were in lower values. The drop in the SMVs of the hydrogels compared with the mMMt could be thus attributed to the non-magnetic behavior of chitosan and HA applied to prepare hydrogels. The SMV was also reported per g of the magnetic matter, so the combination of the non-magnetic ingredients with the magnetic ones resulted in a reduction in the SMV. Moreover, lower SMV corresponded to the amount of the magnetic NPs in the hydrogels. On the other hand, combining chitosan and HA with the mMMt components led to a decline in the amount of the magnetic NPs because of the non-magnetic behavior of these neat biomaterials, which decreased in the SMV. Of note, the magnetic saturation of the Chito/HA-mMMt was enough to discrete it from the hydrogels via an outside magnetic force.

#### TGA study

To investigate the thermal stability of the chitosan and chitosan-based hydrogels, the TGA technique was performed by heating the samples under a nitrogen atmosphere in a range of temperatures, 25–700 ^o^C (Fig. 5b). The neat mMMt, chitosan, and the magnetic carriers accordingly had a weight loss up to 200 ^o^C, which was due to the evaporation of the free and adsorbed water by the samples [48]. The mMMt further showed no special weight loss up to 700 ^o^C, indicating its high thermal stability. About 70 wt% of the Chito/HA was lost from 250 to 700 ^o^C, which was assigned to the chitosan decomposition of chitosan. Introducing the mMMt correspondingly had a significant impact on the thermal stability of the chitosan-based nano-carriers. The weight loss of 60 and 51 wt% also occurred in the Chito/HA-mMMt1 and Chito/HA-mMMt2, respectively. After 300 ^o^C, no significant weight loss was observed in the magnetic nano-carriers, originating from the introduced mMMt with high thermal stability. The weighted residual of the magnetic samples was further found to be more than that of the Chito/HA, representing the high thermal stability of the samples owing to the mMMt introduced for this purpose to attain the magnetic nano-carriers. The difference in the weighted residual of chitosan and the magnetic nano-carriers might be thus related to the amount of the mMMt used to synthesize them. According to the TGA curves, the weighted residual of the Chito/HA-mMMt2 (42.5 wt% more than chitosan) was more than that of the Chito/HA-mMMt1 (32.5 wt% more than chitosan) because of the high amount of the mMMt applied to prepare the Chito/HA-mMMt2.

#### Particle size and zeta-potential study

**Fig. 6:**
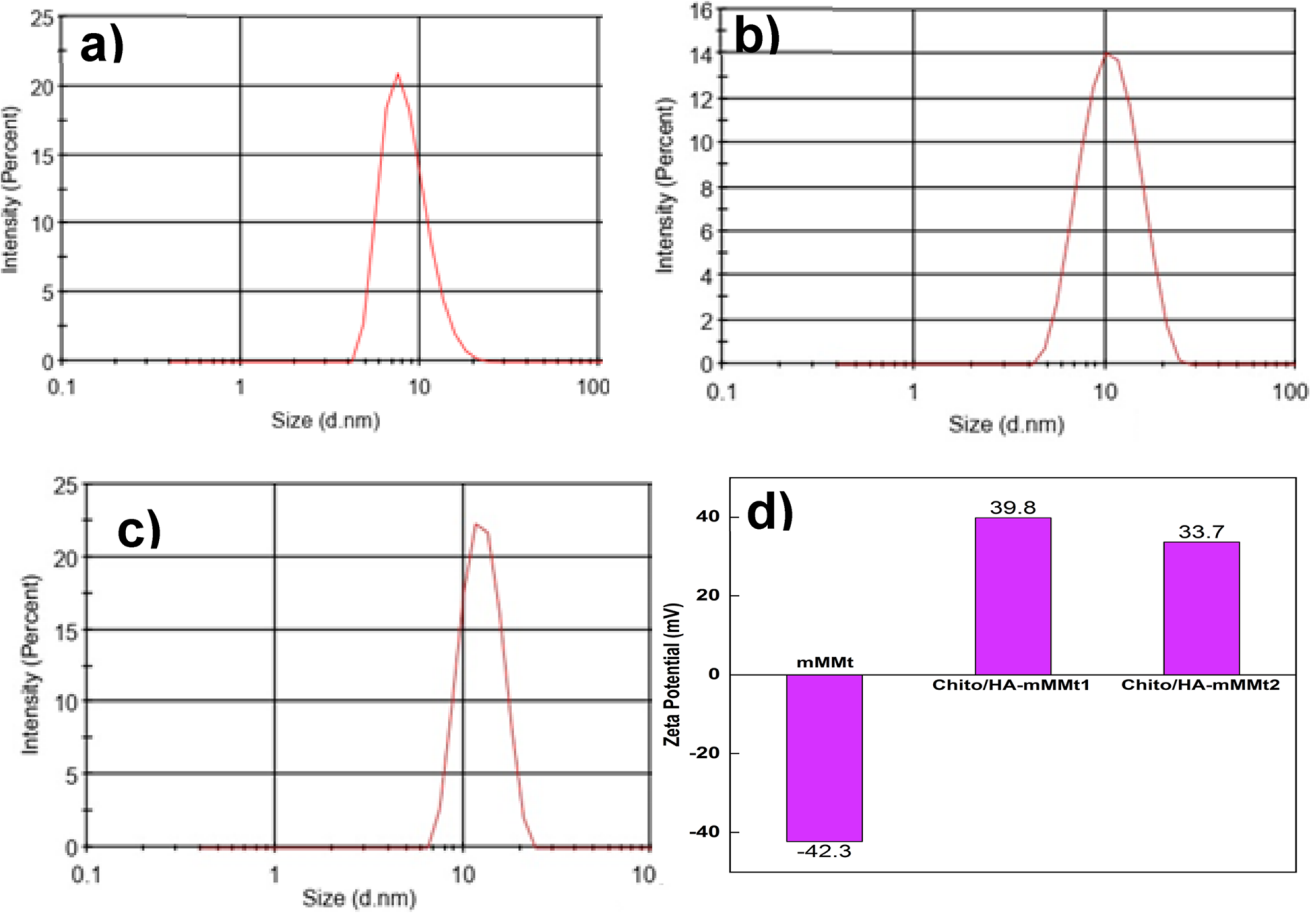
Particle size distribution of **a** mMMt, **b** Chito/HA-mMMt1, **c** Chito/HA-mMMt2, and **d** zeta-potential distribution of samples

The particle size and zeta-potential values of the synthesized hydrogels were explored using the DLS analysis, in which the Stokes-Einstein equation was used to measure the Z-average size of the particles in this instrument. It was assumed that the mMMt NPs were spherical. Figure 6(a to c) shows the mean particle size and zeta-potential of the mMMt, Chito/HA-mMMt1, and Chito/HA-mMMt2.

The smallest particle size and the relatively high net values of the zeta-potential of the samples accordingly revealed the high surface active functionality and stability of the given obtained hydrogels.

Furthermore, the zeta-potential value as indicative of the stability of the nanoparticles and their surface charge was explored. In designing the DDS, it was thus essential to determine the zeta-potential values, so the carriers with high zeta potential were preferred. The high value in the zeta potential could thus stop the aggregation of the NPs. The zeta-potential of the mMMt was further found to be different from the magnetic carriers. The mMMt, Chito/HA-mMMt1, and Chito/HA-mMMt2 also showed the zeta-potential values of -42.3, + 39.8, and + 33.7 mV, respectively (Fig. 6d). The negative value for the mMMt also originated from the negative centers on the MMt. Upon combining the mMMt with chitosan and HA to attain the magnetic NPs, the zeta-potential values tend to elevate and reach positive values. In fact, the results demonstrated the coating effect of the mMMt by the chitosan containing the primary amine (–NH_2_) functional groups. For the Chito/HA-mMMt2, the increase in the mMMt ratio resulted in a reduction in the zeta-potential value, which was associated with the electrostatic interaction between MMt and some –NH_2_ groups of chitosan, leading to a drop in the zeta-potential value.

### Swelling test

As rehydration was necessary to release any coated drug or bioactive material from dried beads, controlling release in DDS could depend on the SF. For this reason, the SF of all hydrogels was done at pH = 7.4 and 5.5 (Table 1). These results showed that the SFs at pH = 7.4 were higher than pH = 5.5 and 1.2. Therefore, the Chito/HA hydrogel was suitable to use in DDS for wound healing. The Chito/HA also displayed high SFs and water uptake percentages compared to other hydrogels because of their high porosity and mechanical properties. On the other hand, the SF rose by entering the water molecules into the hydrogels via the pores. Therefore, these hydrogels consisted of large pores or volumes of water for a long time, thereby providing a wet situation for the wound to be treated quickly. As a result, they could be effective as wound dressings. Moreover, the SF of all hydrogels varied on the basis of the mMMt doses in the hydrogels [49]. The presence of the mMMt-NPs slightly decreases the porosity of the Chito/HA-mMMt hydrogels. The extraordinary porosity of the hydrogels as dressings was thus helpful for absorbing more exudate from the wound surface and reducing wound infection caused by exudate. A wet wound medium could thus help improve the wound and minimize scar formation, while the dressing could be removed without pain.

**Table 1:** The swelling tests and factors of all synthesized hydrogels at pH equal to 7.4 and 5.5

Sample	Dry weight *(W*_*d*_)	pH = 5.5		pH = 7.4	
		Swelled weight (*W*_*s*_) ± SD	Swelling factor (*SF*)	Swelled weight (*W*_*s*_) ± SD	Swelling factor (*SF*)
**Chito/HA**	0.1	0.4258 ± 0.18	3.258	0.4655 ± 0.12	3.655
**Chito/HA-mMMt1**	0.1	0.3108 ± 0.10	2.108	0.3620 ± 0.14	2.620
**Chito/HA-mMMt2**	0.1	0.3059 ± 0.15	2.059	0.3435 ± 0.10	2.435

### Drug release

**Fig. 7:**
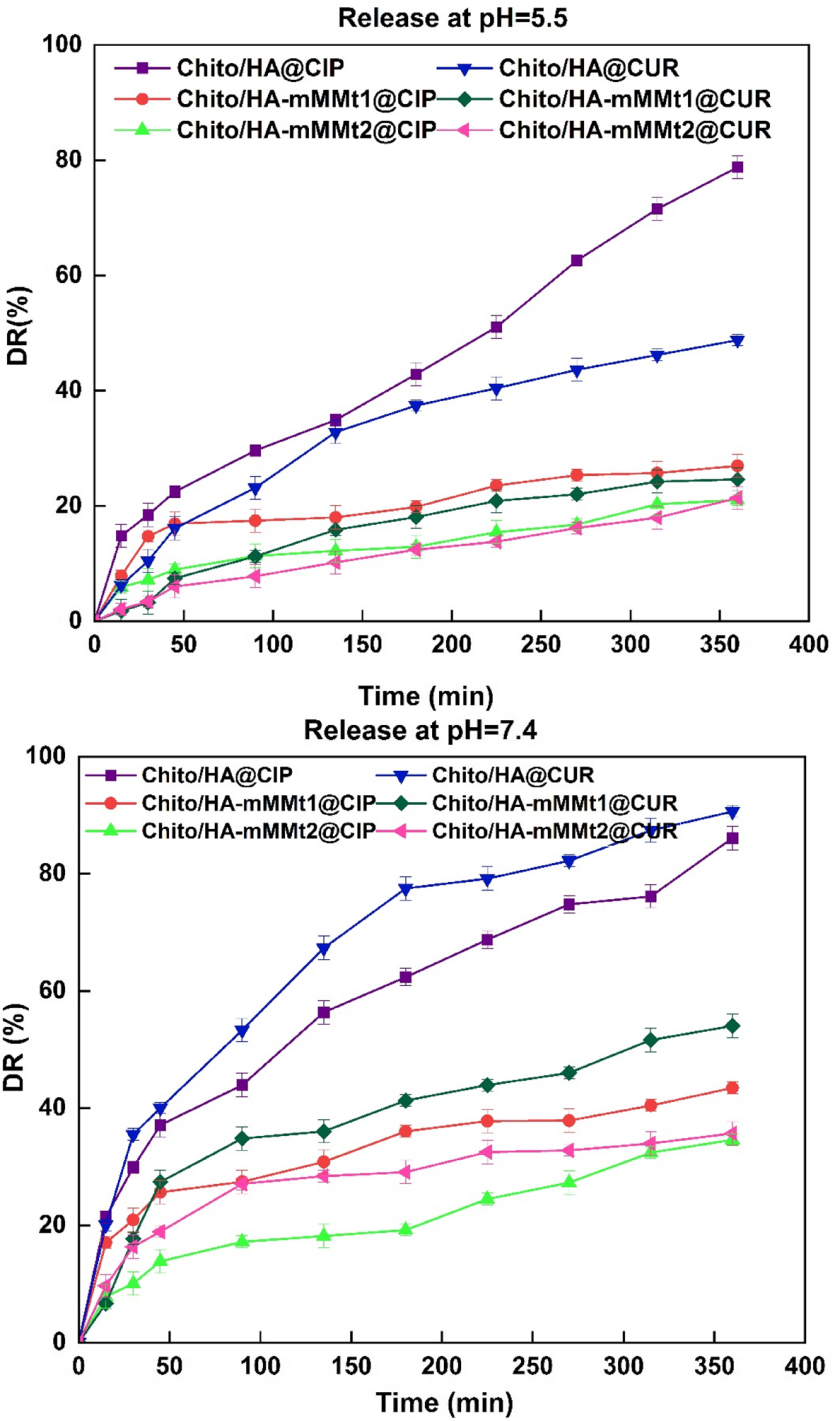
Ciprofloxacin and Curcumin release behavior of Chito/HA, Chito/HA-mMMt1, and Chito/HA-mMMt2 hydrogels at pH = 5.5 and 7.4

The CIP and CUR release from the Chito/HA, Chito/HA-mMMt1, and Chito/HA-mMMt2 hydrogels was examined by measuring their concentrations during the dipping in the media at different pHs (5.5 and 7.4) and at the temperature of 37 ºC. Figure 7 displays the CIP and CUR release from the hydrogels at different pHs (5.5 and 7.4). Not only the pH of the releasing media could have an impact on the amount of CIP and CUR release, but also the amount of MMt could influence it. The increasing amount of the MMt in the hydrogels considerably impacted drug release.

According to the study results, the cumulative release of CUR from the Chito/HA, Chito/HA-mMMt1, and Chito/HA-mMMt2 hydrogels were 89.9, 53.9, and 35.7%, at pH = 7.4 and 48.7%, 24.6%, and 21.8% at pH = 5.5, respectively, after 6 h. The Chito/HA, Chito/HA-mMMt1, and Chito/HA-mMMt2 hydrogels accordingly showed the CIP release of about 78.8, 26.9, and 21.1%, at pH = 5.5 and 86.1%, 43.4%, and 34.6% at pH = 7.4, respectively, after 6 h, possibly due to the electrostatic interactions.

According to the high solubility of CIP under the acid media and the CUR degradation in the base ones, it was expected that the CIP and CUR release at pH = 5.5 would occur higher than that at pH = 7.4. However, the results were inconsistent with the CIP solubility, originating from hydrogels. Figure 7 displays that the release rate is lower at pH = 5.5 than pH = 7.4, because the –CO_2_H groups show more mutual effects with buffer media at pH = 5.5, allowing the network to be tauter; therefore, the entrapped drug molecules hardly get out of the network. Based on the CIP pKa values (pK_a1_=6.18 and pK_a2_=8.8) [50], at pH = 5.5, the amine group on CIP was present in an ionic status (= NH2+), while the carboxylic acid presented in its protonated form (-COOH). At pH = 5.5, free amine pendants were also created by dissociating chitosan and cationic amine groups on it. Furthermore, chitosan was soluble in the acidic media, and its cationic structure could facilitate drug diffusion. The chitosan–NH^3+^ groups could thus boost the diffusion of the drug molecules through electrostatic repulsive force. According to Wu et al. [51], the maximum adsorption of CIP on MMt could occur at pH = 5.5. In fact, the high tendency of the cationic CIP to get adsorbed on the negative centers of the mMMt layers at pH = 5.5 could prevent its easy diffusion into the media. On the other hand, there was a stronger interaction between the group (–CO_2_H) and the negative surface charges in the acidic solution after the CIP adsorption. Additionally, the coating effect of the cross-linked chitosan on the mMMt was not negligible. The protonation of the chitosan biopolymer (pKa ~ 6.5) at pH = 5.5 might thus restrict the diffusion of the cationic CIP from the surface of the mMMt owing to the repulsive forces. The positive charges on the surface of the nano-carriers were also evident from the zeta-potential data. While the mMMt comprised of a negative surface charge after being coated with chitosan, the surface charge could be shifted to a positive charge.

Once the pH of the releasing media increased to 7.4, the physical interactions of the drug and the nano-carriers were changed, resulting in a high release of CIP and CUR. The deprotonation of chitosan at pH = 7.4 also made the cationic nature of coating on mMMt disappear. Thus, the CIP and CUR from the mMMt surface through the chitosan layer could occur quickly without any repulsive forces. While the drug molecules were in the cationic form at pH = 5.5 and 7.4, the dissociation of the carboxylic acid groups to produce anionic carboxylate by maintaining ammonium charges on the drug could make the drug a zwitterion carrying the anionic and cationic charges simultaneously. A repulsive force between the carboxylated groups on the drug and the anionic centers on the mMMt accordingly encouraged the desorption of the drug molecules from the mMMt surface, leading to the drug diffusion through the chitosan layer without any electrostatic interactions.

The decrease in the cumulative drug release from the Chito/HA-mMMt with the high content of the mMMt could be thus related to the interaction between chitosan and the mMMt. It has been also reported that the cross-link density could be augmented due to the MMt used to prepare the hydrogels. The physical interactions through the hydrogen bonding could also lead to a growth in the cross-link points, and subsequently, the pore sizes tended to decrease. The decline in the pore sizes could further result in the restriction in the mobility of CIP to diffuse into the media. The effect of the MMt on the release of the tanshinone IIA (Chemical drug) from chitosan and the chitosan/MMt had been similarly investigated by Luo et al. [52]. The release of tanshinone IIA from the chitosan/MMt microspheres was thus lower than that of the neat chitosan microspheres.

#### Release kinetics

To study the prediction of the mechanism for the drug release, Higuchi and Korsmeyer-Peppas models were utilized with reference to Eqs. 4 and 5, respectively.

4 $$R_r=K_H\sqrt t$$

5 $$R_r=K_{KP}t^n$$

where *R*_*r*_, *K*_*KP*_, and *K*_*H*_ are the drug release rate as well as, the drug release rate constants of the Korsmeyer-Peppas and Higuchi kinetic models, respectively. Moreover, *n* is the diffusion exponent, by which the release mechanism is determined by it. In the Korsmeyer-Peppas model, the *n* value can have different values, of which the values of ≤ 0.45, that between 0.45 and 0.89, and ≥ 0.89 demonstrates the predomination of the Fickian diffusion phenomenon, the anomalous transport (namely, the non-Fickian or diffusion kinetic and polymer relaxation kinetic), and the case-II transport mechanism, respectively. The release mechanism was also affected by the pH of the releasing media, indicating the effect of surface charge on the mechanism of CIP and CUR release. Therefore, the release kinetic was investigated in two media (pH = 5.5 and 7.4).

However, the Higuchi model was defined by plotting the drug (CIP and CUR ) release (DR%) versus the square root of time (t). The outcomes showed a linear relationship for all hydrogels in both media (pH = 7.4 and, 5.5) with R^2^ > 0.95 for CIP and CUR, which suggested the diffusion process for the CIP and CUR release from the hydrogel (Table 2).

**Table 2:** The calculated kinetic parameters of Curcumin and Ciprofloxacin release according to Higuchi and Korsmeyer-Peppas models

		Higuchi model			Korsmeyer-Peppas model	
		K_H_	R^2^	n	K_KP_	R^2^
**For Curcumin loaded in hydrogel**						
**Chito/HA**	**pH = 5.5**	2.78	0.98	0.71	0.81	0.99
	**pH = 7.4**	1.9	0.97	0.89	014	0.98
**Chito/HA-mMMt1**	**pH = 5.5**	1.59	0.98	0.43	0.24	0.99
	**pH = 7.4**	7.68	0.95	0.45	042	0.98
**Chito/HA-mMMt2**	**pH = 5.5**	1.18	0.98	0.41	0.31	0.99
	**pH = 7.4**	1.73	0.95	0.37	0.24	0.99
**For Ciprofloxacin loaded in hydrogel**						
**Chito/HA**	**pH = 5.5**	4.14	0.95	0.53	3.09	0.99
	**pH = 7.4**	4.28	0.99	0.46	7.03	0.98
**Chito/HA-mMMt1**	**pH = 5.5**	1.06	0.95	0.31	4.05	0.99
	**pH = 7.4**	1.98	0.95	0.28	8.16	0.98
**Chito/HA-mMMt2**	**pH = 5.5**	1.01	0.97	0.38	1.97	0.99
	**pH = 7.4**	1.68	0.96	0.44	2.32	0.98

Furthermore, the intercept of *log (DR)* against *log t* plots could determine the *K*_*KP*_ and *n* for the Korsmeyer-Peppas model. Table 2 displays the results of the Korsmeyer-Peppas model fitting. The achieved correlation coefficients of Korsmeyer-Peppas (R^2^) were also higher than 0.98, indicating a linear relationship. The release data of the hydrogels at pH = 5.5 additionally denoted the best fitting to the Korsmeyer-Peppas model, evidenced by the higher R^2^ values (~ 0.99). According to the results in Table 2, the *n* value for the CIP release from the Chito/HA-mMMt1 and Chito/HA-mMMt2 hydrogel and the CUR release from the Chito/HA-mMMt2 were lower than 0.45 at pH = 5.5, showing the release of CIP and CUR from the hydrogels through the Fickain diffusion [53]. Moreover, the R^2^ values for CUR and CIP were higher in the Korsmeyer-Peppas model than in the Higuchi model, indicating the CIP and CUR release from the hydrogels followed by the Korsmeyer-Peppas model. The results accordingly confirmed that the release of CIP and CUR from the hydrogels was dominated via diffusion control [54]. In fact, the release of the drugs from these hydrogels through swelling or dissolution did not occur [55]. Besides, the *n* values at pH = 7.4 (*n* < 0.45) demonstrated the Fickian diffusion of the drug from the hydrogels. Overall, the release of CIP and CUR from the samples through the diffusion process was established by the release kinetic Lajevardi et al. [55] studied the release of cephalexin from the Fe_3_O_4_/silica/MIL/100(Fe)-β/CD. They showed that the mechanism of the cephalexin release from the respective carriers could be altered by changing the pH of the releasing media.

#### Effect of external magnetic field on release

**Fig. 8:**
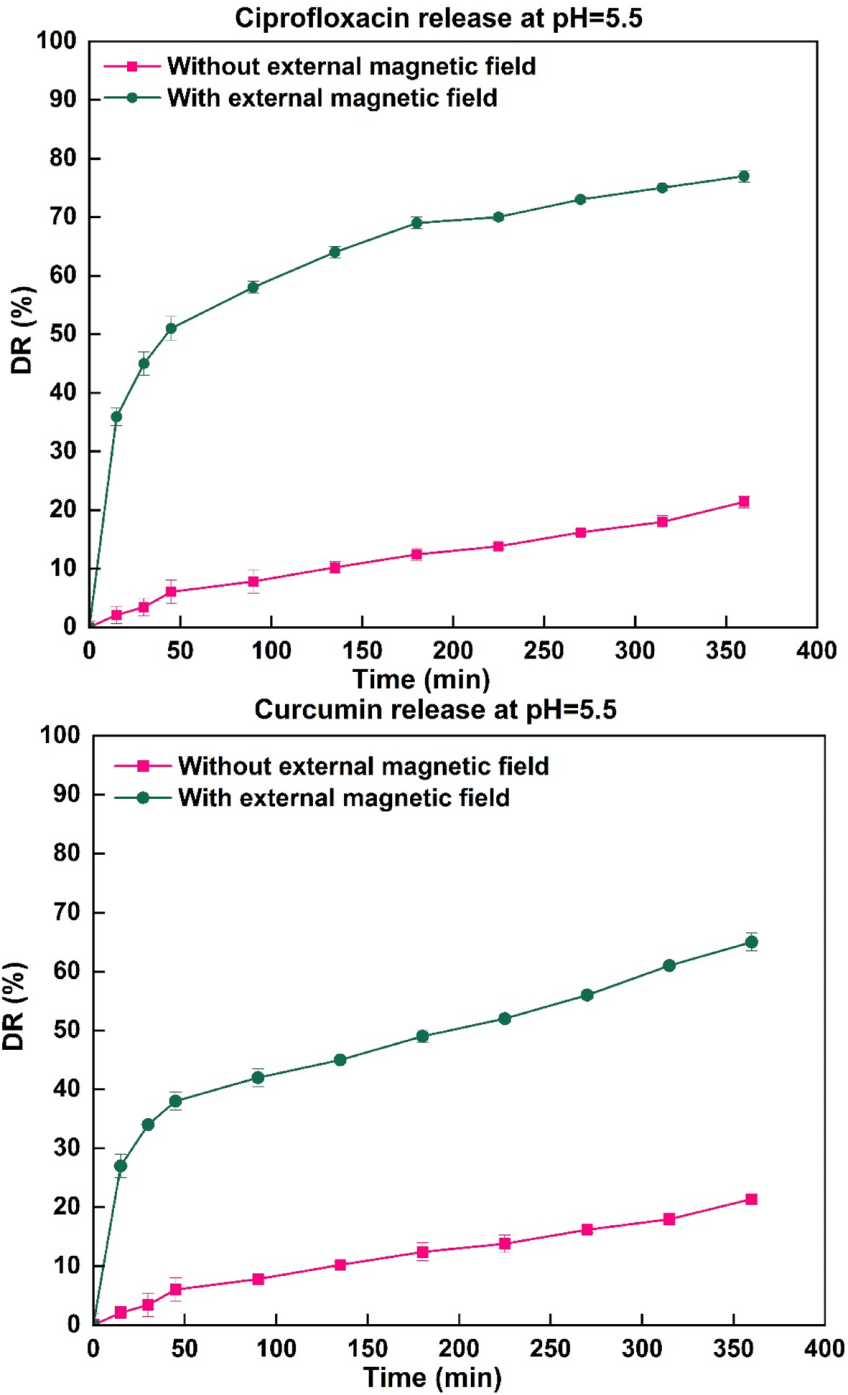
The influence alternating magnetic field on the drug release profiles of Curcumin and Ciprofloxacin from Chito/HA-mMMt2 hydrogel at pH 5.5

Although the main problem facing DDS is the lack of tissue selectivity, the mMMt can deal with this limitation by directing drugs to the targets using an external magnetic field. With regard to the use of the mMMt with magnetic properties, there was an attempt to investigate the release of CIP and CUR from the Chito/HA-mMMt2 by applying an external magnetic field. The impact of the external magnetic field on the CIP and CUR release profiles at pH = 5.5 is illustrated in Fig. 8. Accordingly, the content of the CIP and CUR release under the applied magnetic field was higher than that without it. In the absence of a magnetic field, the cumulative release of CIP and CUR from the Chito/HA-mMMt2 was about 36% and 27% after 15 min, respectively. In contrast, the release of CIP and CUR from the Chito/HA-mMMt2 was accelerated as the magnetic field was applied [41]. Therefore, DDS could be controlled using an external magnetic field to operate the drug release. In this vein, Perera et al. [56] found a comparable outcome that utilizing an external magnetic field had speeded up the release of acetaminophen from the polymer-magnetic composite fibers. Their study was further clarified using the position of the mMMt-NPs in exposing the hydrogels under an external magnetic field. The motion of the mMMt-NPs could thus expand the network because of the relaxation of the polymeric chains [57].

### Antibacterial experiments

**Fig. 9:**
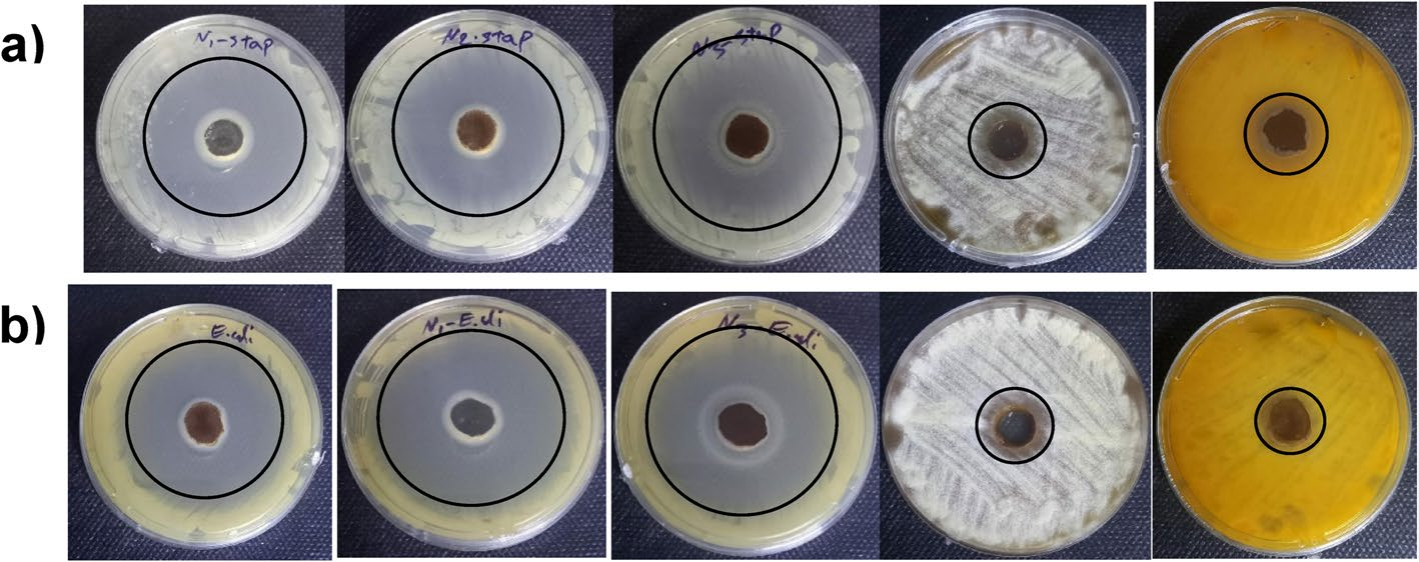
Created zones of inhibition of samples (CUR-CIP loaded Chito/HA, CUR-CIP loaded Chito/HA-mMMt1, CUR-CIP loaded Chito/HA-mMMt2, Chito/HA-mMMt2 without drug, pure CUR-CIP from left to right, against **a** *S. aureus *and **b** *E. coli *incubated at 37 °C for 24 h

The bactericidal activity of the Chito/HA, Chito/HA-mMMt1, and Chito/HA-mMMt2 against Gram-positive and -negative bacteria was further examined here (Fig. 9; Table 3). To reach a better comparison, the effects of CIP and CUR on the antibacterial activity of CIP and CUR-loaded hydrogels were compared with the antibacterial activity of pure chitosan and CIP-CUR. Although the antibacterial activity of chitosan was not negligible, no inhibition zone was observed against Chito/HA-mMMt hydrogel without drug. The bactericidal activity of chitosan also originated from the mobility of chains, which made an inhibition zone appear for bacterial growth. After the cross-linking process using HA and magnetic nanoparticles, the mobility of the chitosan chains became limited, leading to no inhibition zone of bacterial growth. In this line, Li et al. [58] investigated the bactericidal activity of chitosan against Gram-positive and -negative bacteria and found that the cross-linking chitosan scaffold had a significant effect on a decrease in the chitosan bactericidal activity. The CIP and CUR-loaded samples had further exhibited an inhibition zone for both Gram-positive and -negative bacteria, showing the CIP and CUR diffusion from the hydrogels into the media. The inhibition zone for the Chito/HA-mMMt2 sample was also larger as compared with the Chito/HA hydrogels. This was in agreement with the CIP and CUR release behavior of the hydrogels. The Chito/HA-mMMt2 sample also had a higher cumulative release than that of the Chito/HA-mMMt1 one. Furthermore, the mMMt amount affected the antibacterial activities.

**Table 3:** Antibacterial activity of the synthesized hydrogels against S. aureus (Gram-positive) and E. coli (Gram-negative)

No.	Sample	Escherichia coli (mm) ± SD	Staphylococcus aureus (mm) ± SD
**1**	Chito/HA	39 ± 0.1	30 ± 0.2
**2**	Chito/HA-mMMt1	41 ± 0.1	35 ± 0.1
**3**	Chito/HA-mMMt2	43 ± 0.2	36 ± 0.3
**4**	Chitosan	10 ± 0.3	8 ± 0.5
**5**	Curcumin-Ciprofloxacin	11 ± 0.1	8 ± 0.3

### MTT assay

**Fig. 10:**
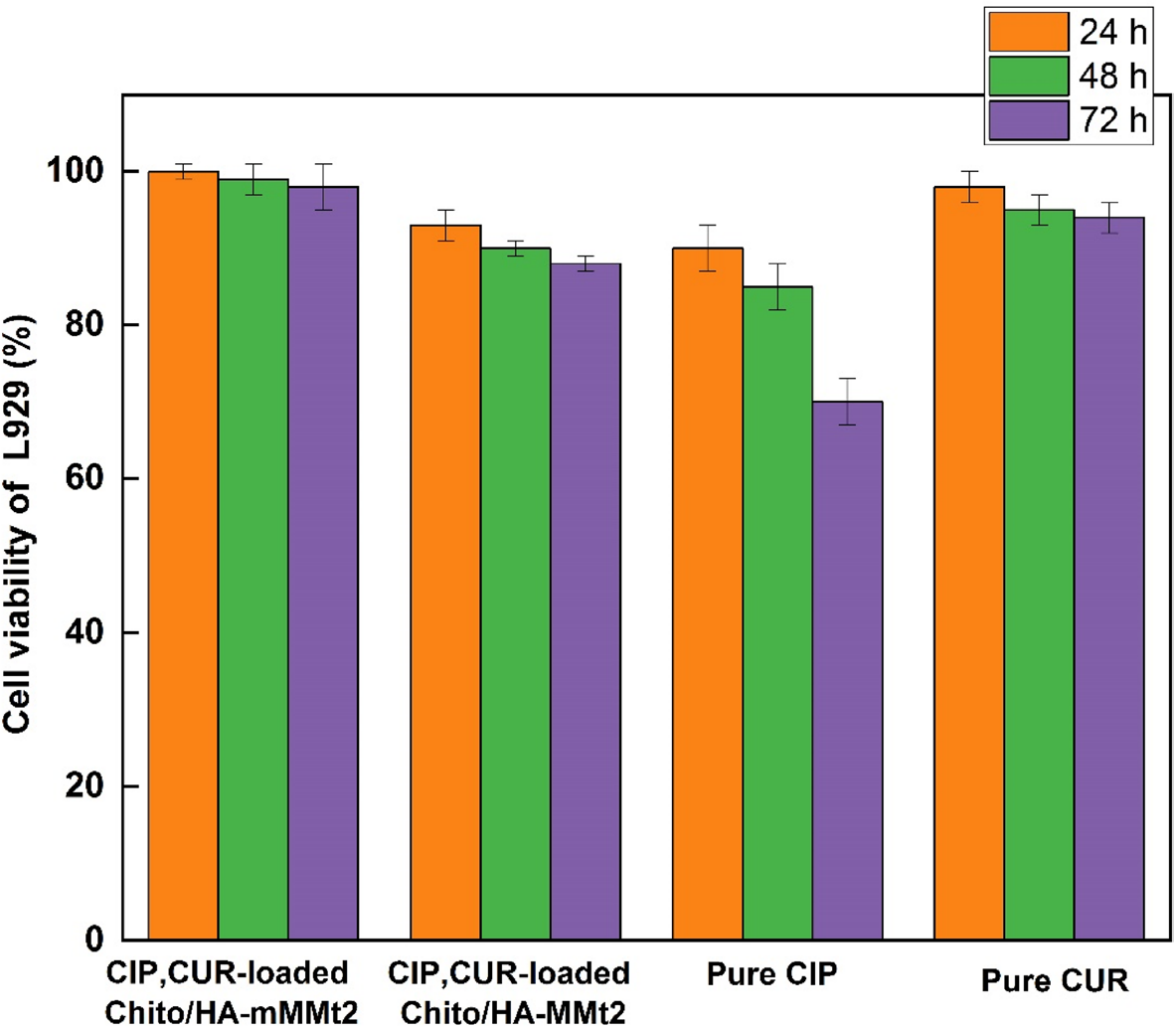
The percentage cell viability of the normal human fibroblastic cells (L929) for 24, 48 and 72 h incubation with pure curcumin, pure ciprofloxacin and CIP,CUR-loaded Chito/HA-mMMt2

In this work, the cell viability percentage for the normal human fibroblastic cells (L929) for 24, 48, and 72 h, incubated with pure CIP and CUR as well as the CIP/CUR-loaded Chito/HA-mMMt2 and CIP/CUR-loaded Chito/HA-MMt2 hydrogels was evaluated using the 3-[4,5-dimethylthiazol-2-yl]-2,5 diphenyl tetrazolium bromide (MTT) assay. Figure 10 displays the cell cytotoxicity results of CIP and CUR as well as the CIP/CUR-loaded Chito/HA-mMMt2 and CIP/CUR-loaded Chito/HA-MMt2 hydrogels. The CIP/CUR-loaded Chito/HA-mMMt2 hydrogel exhibited no noticeable toxicity on the L929 cells because of its biocompatibility. The structure of this hydrogel contained chitosan as a natural polysaccharide, HA, and mMMt, which were non-toxic and biodegradable for enhancing the biocompatibility of DDS. Hence, the Chito/HA-mMMt2 hydrogel could be applied as a harmless bio-DDS for wound healing. Meanwhile, the CIP/CUR-loaded Chito/HA-mMMt2 hydrogel confirmed insignificant cytotoxicity as the pure CIP and CUR. The low release of CIP and CUR from the Chito/HA-mMMt2 hydrogel into the normal cells ultimately led to low cytotoxicity. The MTT assay further presented ~ 70%, ~ 94%, ~ 88% and ~ 98% of the L929 cells viability for CIP and CUR, as well as the CIP/CUR-loaded Chito/HA-MMt2 and CIP/CUR-loaded Chito/HA-mMMt2 hydrogels at the concentration of 16 µg/mL after 72 h, respectively. Cell viability for the pure CIP and CUR was also lower than that of the CIP/CUR-loaded Chito/HA-mMMt2 hydrogel. Besides, the CUR and CIP release from the hydrogel was slower in a controlled manner, and it acted as a membrane. However, the pure CIP or CUR could enter directly into the cells to kill them. Accordingly, it was established that the CIP/CUR-loaded Chito/HA-mMMt2 hydrogel was a non-toxic material for biomedical applications, such as wound healing. Also, the results showed magnetic can improve biocompatibility of Chito/HA hydrogel. Magnetic hydrogel (Chito/HA-mMMt2) did not allow the ciprofloxacin to be released quickly, leading to a reduction in the ciprofloxacin release rate and cytotoxicity.

### Wound healing

**Fig. 11:**
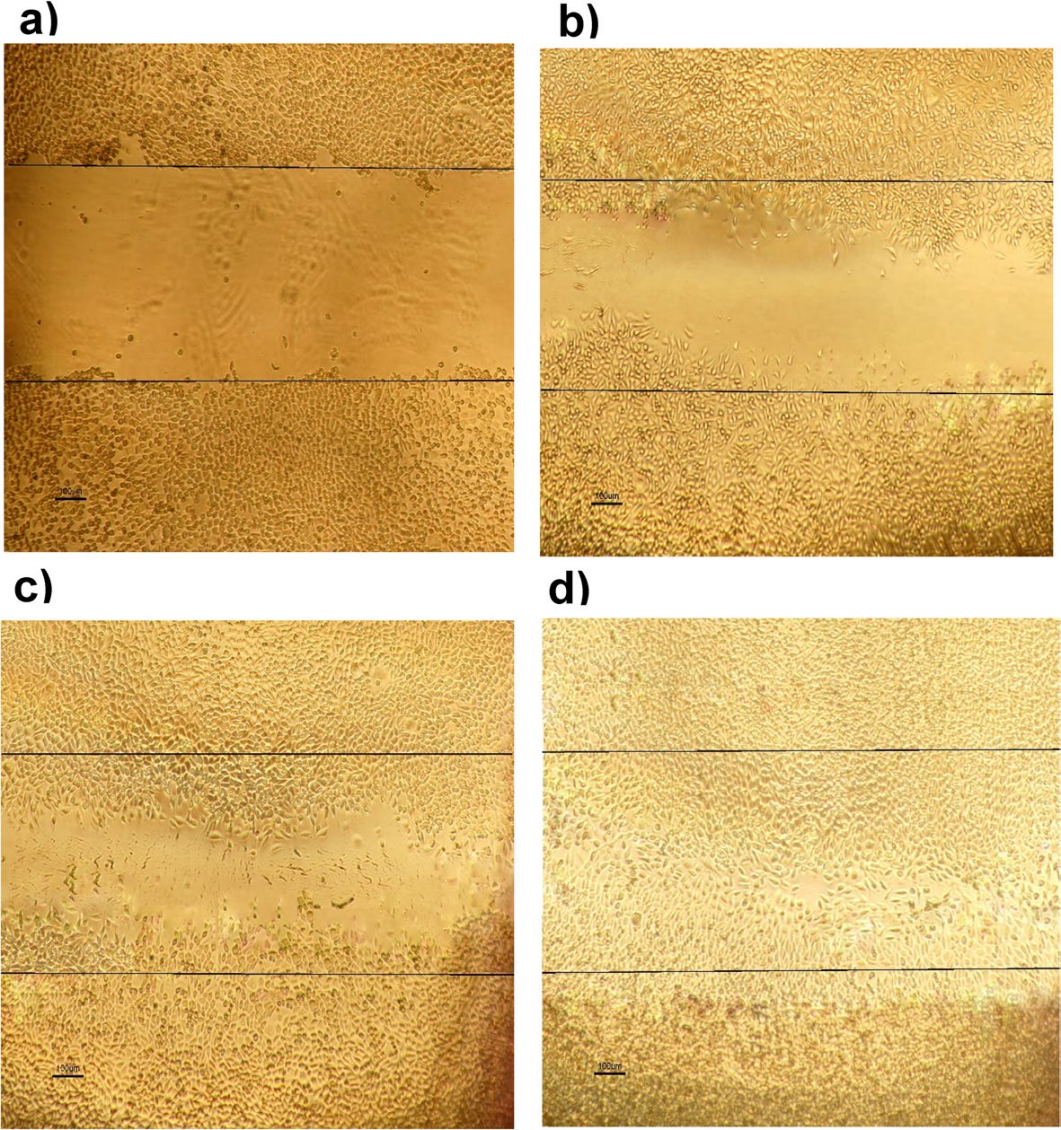
Fluorescent microscope image to estimate wound healing in vitro in the scratch assay using a confluent monolayer of 3T3 fibroblasts (**a**) immediately after the wounding, **b** after 6 h, **c** 12 h, and **d** 48 h incubation

To evaluate the wound healing ability of the Chito/HA-mMMt2 hydrogels, a number of studies have so far reflected on cell growth to investigate the direct or indirect effects of fibroblast motility. As the role of chitosan and HA in wound healing is best known, scratch assays have been typically performed. Moreover, these materials have been applied to motivate the fabrication of matrix proteins in fibroblasts because HA can improve the production of fibronectin and collagen, and synthesize collagenases. As well, chitosan improves granulation tissue formation in skin wounds and wound-bursting strength [59]. Cell migration into the wound was thus detected in response to an artificial injury, as shown in Fig. 11. The incubation time of 48 h accordingly resulted in the highest number of migrated cells in the bare area. However, the number of cells was too high to be measured and clearly distinguished because of the clusters or aggregates.

## Conclusion

In this work, novel pH-sensitive hydrogels were synthesized, including the CIP and CUR loaded in the Chito/HA-mMMt bio-nanocomposite hydrogels. The FT-IR, XRD, SEM, and TEM outcomes also showed the successful synthesis of these hydrogels. The effect of the mMMt amount on the hydrogel swelling, along with the CIP and CUR release and antibacterial activity were further studied. The CIP and CUR release accordingly decreased with increasing mMMt concentration. The release kinetics correspondingly indicated a diffusion mechanism for the CIP and CUR release from these hydrogels. The results of the antibacterial tests additionally revealed that all hydrogels were effective in preventing the growth of *E. coli* and *S. aureus* bacteria. Finally, the MTT assay was fulfilled to examine the in vitro cytotoxicity of the Chito/HA-mMMt hydrogel, indicating no significant cytotoxicity against the L929 cells. According to the scratch assay results, the synthesized hydrogels could be recommended as a DDS for wound healing.

## Data Availability

All data generated or analyzed during this study are included in this published article.

## References

[1] Caló E, Khutoryanskiy VV (2015). Biomedical applications of hydrogels: a review of patents and commercial products. Eur Polym J.

[2] Gun'ko VM, Savina IN, Mikhalovsky SV (2017). Properties of Water Bound in Hydrogels, (in eng). Gel.

[3] Karoyo AH, Wilson LD (2021). A review on the design and hydration properties of natural polymer-based hydrogels. Materials (Basel).

[4] Jafari H, Mahdavinia GR, Kazemi B, Ehrlich H, Joseph Y (2021). Rahimi-Nasrabadi, highly efficient sunitinib release from pH-responsive mHPMC@ Chitosan core-shell nanoparticles. Carbohydr Polym.

[5] Li Y, et al. A pH-sensitive drug delivery system based on folic acid-targeted HBP-modified mesoporous silica nanoparticles for cancer therapy. Colloids Surf A. 2020;590:124470.

[6] Rong SY, Mubarak N, Tanjung FA (2017). Structure-property relationship of cellulose nanowhiskers reinforced chitosan biocomposite films. J Environ Chem Eng.

[7] Udayakumar GP (2021). Biopolymers and composites: properties, characterization and their applications in food, medical and pharmaceutical industries. J Environ Chem Eng.

[8] Azmana M, Mahmood S, Hilles AR, Rahman A, Arifin MAB, Ahmed S (2021). A review on chitosan and Chitosan-based bionanocomposites: promising material for combatting global issues and its applications. J Environ Chem Eng.

[9] Ardean C (2021). Factors influencing the antibacterial activity of Chitosan and Chitosan modified by functionalization. Int J Mol Sci.

[10] Ruiz-Pulido G, Quintanar-Guerrero D, Serrano-Mora LE, Medina DI (2022). Triborheological Analysis of Reconstituted Gastrointestinal Mucus/Chitosan: TPP nanoparticles System to study Mucoadhesion Phenomenon under different pH conditions. Polym.

[11] Jaiswal S, Dutta P, Kumar S, Chawla R (2021). Chitosan modified by organo-functionalities as an efficient nanoplatform for anti-cancer drug delivery process. J Drug Delivery Sci Technol.

[12] Rostami E (2020). Progresses in targeted drug delivery systems using chitosan nanoparticles in cancer therapy: a mini-review. J Drug Delivery Sci Technol.

[13] Heragh BK, Javanshir S, Mahdavinia GR, Jamal MRN (2021). Hydroxyapatite grafted chitosan/laponite RD hydrogel: evaluation of the encapsulation capacity, pH-responsivity, and controlled release behavior. J Environ Chem Eng.

[14] Md S (2018). Nano-carrier enabled drug delivery systems for nose to brain targeting for the treatment of neurodegenerative disorders. J Drug Delivery Sci Technol.

[15] Vaze N (2019). Inactivation of common hospital acquired pathogens on surfaces and in air utilizing engineered water nanostructures (EWNS) based nano-sanitizers, Nanomed. Nanotechnol Biolo Med.

[16] Najafloo R, Behyari M, Imani R, Nour S (2020). A mini-review of Thymol incorporated materials: applications in antibacterial wound dressing. J Drug Delivery Sci Technol.

[17] Shrestha S, Kishen A (2019). Temporal-controlled bioactive molecules releasing core-shell nano-system for tissue engineering strategies in endodontics. Nanomed Nanotechnol Biolo Med.

[18] Chen F, Shi S (2014). Principles of tissue Engineering.

[19] Rasool A, Ata S, Islam A (2019). Stimuli responsive biopolymer (chitosan) based blend hydrogels for wound healing application. Carbohydr Polym.

[20] Saadat S, Rawtani D, Braganza V (2022). Antimicrobial activity of chitosan film containing nanocomposite of Trachyspermum ammi (ajwain) seed oil loaded Halloysite nanotubes against foodborne pathogenic microorganisms. Appl Clay Sci.

[21] de Lima PHC, Tavares AA, de Lima Silva SM, de Moura MR, Aouada FA, Grillo R (2022). Recent advances on nanohybrid systems constituting clay–chitosan with organic molecules – A review. Appl Clay Sci.

[22] Qu B, Luo Y (2021). A review on the preparation and characterization of chitosan-clay nanocomposite films and coatings for food packaging applications. Carbohydr Polym Technol Appl.

[23] Wei W (2021). Advanced hydrogels for the repair of cartilage defects and regeneration. Bioact Mater.

[24] Sadjadi S, Abedian-Dehaghani N, Heydari A, Heravi MM (2023). Chitosan bead containing metal–organic framework encapsulated heteropolyacid as an efficient catalyst for cascade condensation reaction. Sci Rep.

[25] Zhou Y (2018). Photopolymerized maleilated chitosan/methacrylated silk fibroin micro/nanocomposite hydrogels as potential scaffolds for cartilage tissue engineering. J Environ Chem Eng.

[26] Liu M (2017). Injectable hydrogels for cartilage and bone tissue engineering. Bone Res.

[27] Jiang X (2022). Chitosan/clay aerogel: Microstructural evolution, flame resistance and sound absorption. Appl Clay Sci.

[28] Yamashita Y, Ohzuno Y, Saito Y, Fujiwara Y, Yoshida M, Takei T (2023). Autoclaving-triggered hydrogelation of Chitosan-Gluconic acid Conjugate Aqueous Solution for Wound Healing. Gels.

[29] Yao Z-Y (2021). Versatile strategies for bioproduction of hyaluronic acid driven by synthetic biology. Carbohydr Polym.

[30] Arshad R (2021). A hyaluronic acid functionalized self-nano-emulsifying drug delivery system (SNEDDS) for enhancement in ciprofloxacin targeted delivery against intracellular Infection. Nanomat.

[31] Jayrajsinh S, Shankar G, Agrawal YK, Bakre L (2017). Montmorillonite nanoclay as a multifaceted drug-delivery carrier: a review. J Drug Delivery Sci Technol.

[32] Wang S, Jing Y (2017). Effects of formation and penetration properties of biodegradable montmorillonite/chitosan nanocomposite film on the barrier of package paper. Appl Clay Sci.

[33] Hosseinzadeh H, Zoroufi S, Mahdavinia GR (2015). Study on adsorption of cationic dye on novel kappa-carrageenan/poly (vinyl alcohol)/montmorillonite nanocomposite hydrogels. Polym Bull.

[34] Bortolin A, Serafim AR, Aouada FA, Mattoso LH, Ribeiro C (2016). Macro-and micronutrient simultaneous slow release from highly swellable nanocomposite hydrogels. J Agric Food Chem.

[35] He F, Zhou Q, Wang L, Yu G, Li J, Feng Y (2019). Fabrication of a sustained release delivery system for pesticides using interpenetrating polyacrylamide/alginate/ montmorillonite nanocomposite hydrogels. Appl Clay Sci.

[36] Jaberifard F, Ghorbani M, Arsalani N, Mostafavi H (2022). A novel insoluble film based on crosslinked-starch with gelatin containing ZnO-loaded halloysite nanotube and bacterial nanocellulose for wound healing applications. Appl Clay Sci.

[37] Petkovic M, Sørensen AE, Leal EC, Carvalho E, Dalgaard LT (2020). Mechanistic actions of microRNAs in diabetic wound healing. Cells.

[38] Massaro M (2021). Ciprofloxacin carrier systems based on hectorite/halloysite hybrid hydrogels for potential wound healing applications. Appl Clay Sci.

[39] Sayyar Z, Jafarizadeh-Malmiri H (2020). Process intensification for curcumin nanodispersion preparation using subcritical water optimization and characterization. Chem Eng Process.

[40] Kumari A (2022). Wound-Healing effects of Curcumin and its nanoformulations: a Comprehensive Review. Pharm.

[41] Jafari H, Atlasi Z, Mahdavinia GR, Hadifar S, Sabzi M (2021). Magnetic κ-carrageenan/chitosan/montmorillonite nanocomposite hydrogels with controlled sunitinib release. Mater Sci Eng.

[42] Bodnar M, Hartmann JF, Borbely J (2005). Preparation and characterization of Chitosan-based nanoparticles. Biomacromol.

[43] Olad A, Pourkhiyabi M, Gharekhani H, Doustdar F (2018). Semi-IPN superabsorbent nanocomposite based on sodium alginate and montmorillonite: reaction parameters and swelling characteristics. Carbohydr Polym.

[44] Uranga J, Puertas AI, Etxabide A, Dueñas MT, Guerrero P (2019). De La Caba, citric acid-incorporated fish gelatin/chitosan composite films. Food Hydrocolloids.

[45] Nikzad S, Amooey AA, Alinejad-Mir A (2021). High effective removal of diazinon from aqueous solutions using the magnetic tragacanth-montmorillonite nanocomposite: isotherm, kinetic, and mechanism study. Environ Sci Pollut Res.

[46] Larraza I, López-Gónzalez M, Corrales T, Marcelo G (2012). Hybrid materials: magnetite–polyethylenimine–montmorillonite, as magnetic adsorbents for cr (VI) water treatment. J Colloid Interface Sci.

[47] Mahdavinia GR, Hasanpour S, Behrouzi L, Sheykhloie H (2016). Study on adsorption of Cu (II) on magnetic starch-g-polyamidoxime/montmorillonite/Fe3O4 nanocomposites as novel chelating ligands. Starch-Stärke.

[48] Minisy IM, Salahuddin NA, Ayad MM (2021). Adsorption of methylene blue onto chitosan–montmorillonite/polyaniline nanocomposite. Appl Clay Sci.

[49] Altunkaynak F, Okur M, Saracoglu N (2022). Controlled release of paroxetine from chitosan/montmorillonite composite films. J Drug Delivery Sci Technol.

[50] Sun J (2002). Determination of lipophilicity of two quinolone antibacterials, ciprofloxacin and grepafloxacin, in the protonation equilibrium. Eur J Pharm Biopharm.

[51] Wu Q, Li Z, Hong H, Yin K, Tie L (2010). Adsorption and intercalation of ciprofloxacin on montmorillonite. Appl Clay Sci.

[52] Luo C, Yang Q, Lin X, Qi C, Li G (2019). Preparation and drug release property of tanshinone IIA loaded chitosan-montmorillonite microspheres. J Environ Chem Eng.

[53] Anirudhan T, Christa J (2020). Temperature and pH sensitive multi-functional magnetic nanocomposite for the controlled delivery of 5-fluorouracil, an anticancer drug. J Drug Delivery Sci Technol.

[54] Rezk AI, Obiweluozor FO, Choukrani G, Park CH, Kim CS (2019). Drug release and kinetic models of anticancer drug (BTZ) from a pH-responsive alginate polydopamine hydrogel: towards cancer chemotherapy. J Environ Chem Eng.

[55] Lajevardi A, Sadr MH, Yaraki MT, Badiei A, Armaghan M (2018). A pH-responsive and magnetic Fe 3 O 4@ silica@ MIL-100 (Fe)/β-CD nanocomposite as a drug nanocarrier: loading and release study of cephalexin. New J Chem.

[56] Perera AS, Zhang S, Homer-Vanniasinkam S, Coppens M-O, Edirisinghe M (2018). Polymer–magnetic composite fibers for remote-controlled drug release. ACS Appl Mater Interfaces.

[57] Hu X, Wang Y, Zhang L, Xu M, Zhang J, Dong W (2018). Magnetic field-driven drug release from modified iron oxide-integrated polysaccharide hydrogel. J Environ Chem Eng.

[58] Li Y (2020). Drug-free and non-crosslinked chitosan scaffolds with efficient antibacterial activity against both Gram-negative and Gram-positive bacteria. Carbohydr Polym.

[59] Vigani B, Rossi S, Sandri G, Bonferoni MC, Caramella CM, Ferrari F (2019). Hyaluronic acid and Chitosan-based nanosystems: a new dressing generation for wound care, Expert Opin. Drug Delivery.

